# The change of coagulation profile in two-staged arthroplasty for periprosthetic joint infection patients: a retrospective cohort study

**DOI:** 10.1186/s13018-021-02477-4

**Published:** 2021-05-18

**Authors:** Hao Li, Rui Li, L. L. Li, Wei Chai, Chi Xu, Jiying Chen

**Affiliations:** 1 Medical School of Chinese PLA, Beijing, People’s Republic of China; 2 Department of Orthopedic Surgery, The First Medical Center, Chinese PLA General Hospital, 28 Fuxing Road, Beijing, People’s Republic of China

**Keywords:** Periprosthetic joint infection, Total joint arthroplasty, Two-staged arthroplasty, Coagulation profile, Activated partial thromboplastin time

## Abstract

**Aims:**

Periprosthetic joint infection (PJI) is a serious complication of total joint arthroplasty. We performed a retrospective cohort study to evaluate (1) the change of coagulation profile in two-staged arthroplasty patients and (2) the relationship between coagulation profile and the outcomes of reimplantation.

**Method:**

Between January 2011 and December 2018, a total of 202 PJI patients who were operated on with two-staged arthroplasty were included in this study initially. This study continued for 2 years and the corresponding medical records were scrutinized to establish the diagnosis of PJI based on the 2014 MSIS criteria. The coagulation profile was recorded at two designed points, (1) preresection and (2) preimplantation. The difference of coagulation profile between preresection and preimplantation was evaluated. Receiver operating characteristic curves (ROC) were used to evaluate the diagnostic efficiency of the coagulation profile and change of coagulation profile for predicting persistent infection before reimplantation.

**Results:**

The levels of APTT, INR, platelet count, PT, TT, and plasma fibrinogen before spacer implantation were significantly higher than before reimplantation. No significant difference was detected in the levels of D-dimer, ACT, and AT3 between the two groups. The AUC of the combined coagulation profile and the change of combined coagulation profile for predicting persistent infection before reimplantation was 0.667 (95% CI 0.511, 0.823) and 0.667 (95% CI 0.526, 0.808), respectively.

**Conclusion:**

The coagulation profile before preresection is different from before preimplantation in two-staged arthroplasty and the coagulation markers may play a role in predicting infection eradication before reimplantation when two-stage arthroplasty is performed.

**Level of evidence:**

Level III, diagnostic study.

**Supplementary Information:**

The online version contains supplementary material available at 10.1186/s13018-021-02477-4.

## Introduction

Total joint arthroplasty (TJA) has been one of the most successful surgeries during the last century. Patients with advanced joint diseases can achieve relief of pain and functional recovery after this surgery. However, periprosthetic joint infection (PJI) is a disastrous complication after total joint arthroplasty and often indicates unfavorable outcomes [1, 2]. Although two-staged arthroplasty is a preferred treatment for chronic PJI, it is still difficult to predict persistent infection before reimplantation [3] and inappropriate implantations can lead to treatment failure. A failure of two-stage reimplantation can result in more disastrous complications and the need for further arthroplasty, arthrodesis, and/or amputation [4].

The pathogens of PJI are known to impair their hosts by endotoxin and exotoxin which can stimulate immune cells to produce various cytokines such as IL-6, TNF, and IL-6 [5–7]. These secreted cytokines may disrupt normal coagulation cascade and cause abnormal coagulation profiles in PJI patients. Some studies revealed that the coagulation profile of PJI patients was different from that of non-PJI patients. It suggests that PJI patients suffer from abnormal coagulation. Theoretically, the subclinical abnormal coagulation can increase the risk of epidural hematoma formation and impair incision healing after surgery [6–8]. However, a review of the literatures suggests that the studies about the relationship between the coagulation system and the clinical outcomes of PJI are limited [9–12]. We hold the opinion that the effects of pathogens on the coagulation system may disappear, and the corresponding abnormal coagulation profile may return to normal when the pathogens are eradicated because the endotoxin and exotoxin produced by pathogens are removed.

Based on what was mentioned, we propose a hypothesis that the coagulation profile of PJI patients before reimplantation is different from before spacer implantation when the infection was controlled and the changed coagulation profile can play a role in predicting persistent infection before reimplantation. Some studies revealed that plasma fibrinogen can play a role in predicting persistent infection before reimplantation in two-stage exchange arthroplasty for PJI. However, there is still a lack of comprehensive studies evaluating the change of coagulation profiles in two-staged exchange arthroplasty and the association between the coagulation profile before reimplantation, and the outcomes of two-stage exchange arthroplasty [13, 14].

In a bid to address the problems mentioned above, we performed a retrospective cohort study with at least 2 years of follow-up to evaluate (1) the change of the coagulation profile in two-staged arthroplasty PJI patients and (2) the use of coagulation profile in predicting persistent infection before reimplantation.

## Materials and methods

### Inclusion and exclusion criteria

Institutional review board approval was obtained prior to the commencement of this study, and then, a total of 202 PJI patients treated with two-staged arthroplasty are included in this study. All of the patients were performed with two-staged arthroplasty for PJI treatment in our center. The inclusion criteria were (1) patients diagnosed with PJI by 2014 MSIS criteria and (2) PJI patients treated with 2-staged arthroplasty.

**Fig. 1:**
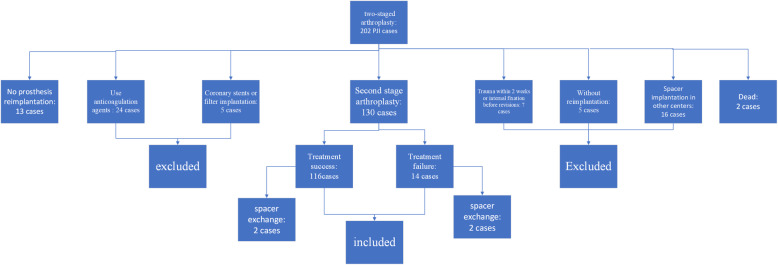
Patients’ details

The exclusion criteria were (1) patients who were exposed to anticoagulation agents within 2 weeks before reimplantation and preresection; (2) periprosthetic fracture; (3) periprosthetic dislocation; (4) patients who received coronary stents, filter implantation, and internal fixation implantation; (5) patients who did not receive new prosthesis reimplantation after spacer implantation; and (6) patients who receive spacer implantation in other joint centers. A total of 130 patients were included in this study; the details are shown in Fig. 1. The medical records of PJI patients were scrutinized and the diagnosis of PJI was based on the 2014 MSIS criteria.

Furthermore, the following data was collected to reflect the coagulation profile of PJI patients: APTT, TT, INR, prothrombin time (PT), plasma D-dimer, plasma fibrinogen, plasma Ca+, platelet count, antithrombin 3, and prothrombin activity (PTA).

### Treatment protocol

According to the institutional standard of two-stage arthroplasty, the two-stage arthroplasty is performed by several surgeons. During the first stage of two-stage arthroplasty, all implanted prostheses are removed, then followed by an extensive debridement and irrigation; betadine and hydrogen peroxide is used separately for the irrigation. Irrigate the infected joint three times accompanied by pulsed washes with saline. Then, an articular antibiotic-loaded cement spacer (polymethylmethacrylate, PMMA) is inserted. The antibiotics added to the bone cement are selected based on the preoperative culture result and antibiotic sensitivity test. In the case of culture-negative PJI, an articular antibiotic-loaded cement spacer containing 8g vancomycin and 4g meropenem per 40g bone cement is then implanted.

After the spacer implantation, 6 weeks of IV antibiotics is administrated based on tests of antibiotic sensitivity, followed by 6 weeks of po. antibiotics until the infection is controlled. An antibiotic holiday of at least 2 weeks is stipulated before reimplantation.

The timing of reimplantation was based on the following criteria:

1. No clinical symptoms and signs of joint infection such as fistula, pain, and effusions
2. A gradual decrease in plasma ESR and CRP after discontinuation of antibiotic administration

### Definition of treatment failure and treatment success

All PJI patients were checked up upon for 2 years after reimplantation and the outcomes of follow-up were used to evaluate the prognosis of 2-staged reimplantation. Patients were checked on at 3 months, 6 months, 12 months, and 24 months after surgery. Follow-up visits include outpatient and remote follow-up visits. Imaging data (radiographs and pictures of the healing of the postoperative incision) for remote follow-up visits were taken locally and the imaging data was collected in the form of electronic photos. During the follow-up period after reimplantation, if PJI was suspected, a joint aspiration and subsequent synovial fluid analysis was performed.

Treatment failure was defined as:

1. Re-infection after reimplantation based on the MSIS criteria
2. Two identical pathogens revealed by at least two intraoperative periprosthetic cultures at reimplantation

The Delphi-based criteria were used to define treatment success; they were summarized as:

1. A healed incision without fistula, pain, effusions, and recurrence of infection caused by the same pathogen.
2. No surgery was performed on the infected joint after reimplantation.
3. No mortality due to PJI.

### Statistical analysis

The variables were divided into continuous variables and dichotomous data based on the types of data. A normal distribution test was used to evaluate the distribution of continuous variables. The continuous variables were described as means if the normal distribution was achieved. Otherwise, corresponding medians were calculated. Rank sum test and Student t-test were used to detect the difference if the corresponding applicable conditions were met. Dichotomous data were described as frequencies and compared by chi-squared test subsequently. P<0.05 indicates statistical significance. Paired tests were used to compare the difference of coagulation profile between preresection and preimplantation. Receiver operating characteristic curves (ROC) were used to evaluate the efficiency of the coagulation profile in predicting persistent infection before reimplantation. Logistic regression was used to build diagnostic models based on the coagulation profile. Yonder’s index was used to identify optimal cut-off. SPSS (IBM; version 26.0) was used to perform statistical analysis.

## Results

### Demographic characteristics

The median ages in the treatment success group and the treatment failure group were 63 and 60 years, respectively. The median BMI in the success group and failure groups were 23.95 kg/m^2^ and 28.72 kg/m^2^, respectively. The percentage of females in the success group and failure group was 58.62% and 35.71%, respectively. The percentage of the knee in the success group and failure group was 42.24% and 57.14%, respectively. The percentage of patients with inflammatory joint diseases in the success group and failure group was 5.17% and 7.14%, respectively. The details of the demographic characteristics are summarized in Table 1.

**Table 1:** Demographic characteristics

	***Treatment success*** ***N=116***	***Treatment failure*** ***n=14***	***P values***
***Age***	63 (27, 84)	60 (24, 76)	0.942
***Female (n, %)***	68, *58.62%*	5, *35.71%*	0.103
***Knee (n, %)***	49, *42.24%*	8, *57.14%*	0.288
BMI*	23.95 (16.6, 36.49)	28.72 (22.1, 28.7)	0.108
***ASA***			
***2***	63, *54.31%*	10, *71.42%*	0.223
***3***	6, *5.17 %*	0	1
***4***	0	0	1
***Liver disease(n,%)***	3, *2.59%*	0	1
***Kidney disease(n,%)***	1, *0.86%*	0	1
***Heart disease(n,%)***	9, *7.76%*	1, *7.14%*	1
***DM(n,%)***	19, *16.38%*	0	1
***Inflammatory joint diseases (n,%)***	6, *5.17%*	1, 7.14*%*	1
Spacer interval(day)*	120.5 (32, 535)	144 (52, 308)	0.47
***Causative pathogens***			
***CNS***	50, *43.10%*	2, *14.29%*	0.038
***Staphylococcus aureus***	7, *6.03%*	4, *28.57%*	0.019
***Enterococcus spp.***	5, *4.31%*	0	1
***Streptococcus spp.***	8, *6.90%*	1, *7.14%*	1
***Gram-negative bacteria***	10, *8.62%*	4, *28.57%*	0.045
***Fungi***	5, *4.31%*	0	1
***Other pathogens***	6, *5.26%*	1, *7.14%*	0.559
***Culture negative***	40, *34.48%*	3, *21.43%*	0.385

*Values were given as medians (minimum, maximum)

### The change of coagulation profile in PJI two-staged arthroplasty

The levels of APTT, INR, platelet count, PT, and plasma fibrinogen before spacer implantation (group A) were significantly higher than before reimplantation (group B). No significant difference was detected in the levels of plasma D-dimer and AT3 between the two groups. The median APTT in group A was 38.3s and 35.9s in group B. The median PT in group A was 13.5s and 13.3s in group B. The median TT in group A was 15.9s and 15.85s in group B. The median INR in group A was 1.04 and 1.02 in group B. The median plasma fibrinogen in group A was 4.86g/L and 3.36g/L in group B. The median platelet count in group A was 277109/dl and 195 109/dl in group B. The median plasma Ca in group A was 2.23 mmol/L and 2.26 mmol/L in group B. The details about the change of coagulation profiles are shown in Table 2. No significant difference was revealed between the coagulation profile in the treatment success group than that of in the treatment failure group. The details of the coagulation profile before reimplantation (treatment failure versus treatment success) are shown in Table 3.

**Table 2:** The change of coagulation profile in PJI two-staged arthroplasty

***Coagulation profile***	***Preresection***	***Preimplantation***	***P values***
***APTT(s)***	38.3 (18.1, 62.9)	35.9 (23.7, 51.6)	<0.001*
***PT(s)***	13.5 (11.7, 39)	13.3 (11.9, 16.8)	0.016*
***TT(s)***	15.9 (14, 21.1)	15.85 (4, 19.1)	0.183
***INR***	1.04 (0.86, 1.97)	1.02 (0.87, 1.38)	0.003*
Plasma fibrinogen (g/L)**	4.86 (2.30, 8.08)	3.36 (1.21, 8.9)	<0.001*
***Plasma Ca (mmol/L)***	2.23 (1.2, 2.27)	2.26 (1.9, 2.58)	0.158
***AT3 (%)***	86 (2.28, 125)	89 (2.88, 129)	0.306
***D-dimer (ug/ml)***	1.57 (0.2, 6.39)	1.49 (0.17, 12.27)	0.577
***Platelet count (10***^***9***^***/dl)***	277 (262, 292)	207 (196, 218)	<0.001*

*P<0.05

Values were given as medians (minimum, maximum)

*values were given as means (95% CI)

**Table 3:** The difference of coagulation profile between controlled infection group and persistent infection group before reimplantation

***Coagulation profile***	***Treatment success***	***Treatment failure***	***P value***
***APTT***^a^	35.3 (23.7, 46.1)	37.05 (33.8, 45.5)	0.755
***PT***	13.4 (11.9, 16.8)	13.2 (12.3, 15.1)	0.188
***TT***	15.8 (4, 18.6)	15.1 (13.5, 16.8)	0.827
***INR***	1.02 (0.89, 1.38)	1.01 (0.93, 1.19)	0.106
***Plasma fibrinogen***	3.19 (1.21, 6.1)	3.88 (3.12, 6.44)	0.822
***Plasma Ca***^a^	2.22 (1.9, 2.51)	2.22 (2.01, 2.39)	0.583
***AT3***	91 (2.88, 129)	90 (77, 116)	0.552
***D-dimer***	1.49 (0.17, 11.68)	1.1 (0.69, 4.72)	0.668
***Platelet count***	196.23 (175.95, 216.51)	195 (152.48, 237.52)	0.468
***ESR***	12 (2, 36)	11 (4, 30)	0.706
***CRP***	0.298 (0.05, 4.46)	0.71 (0.1, 2.57)	0.220

Values were given as medians (minimum, maximum)

^a^Values were given as means (95% CI)

### The association between the coagulation profile and treatment outcomes of two-staged arthroplasty

**Fig. 2:**
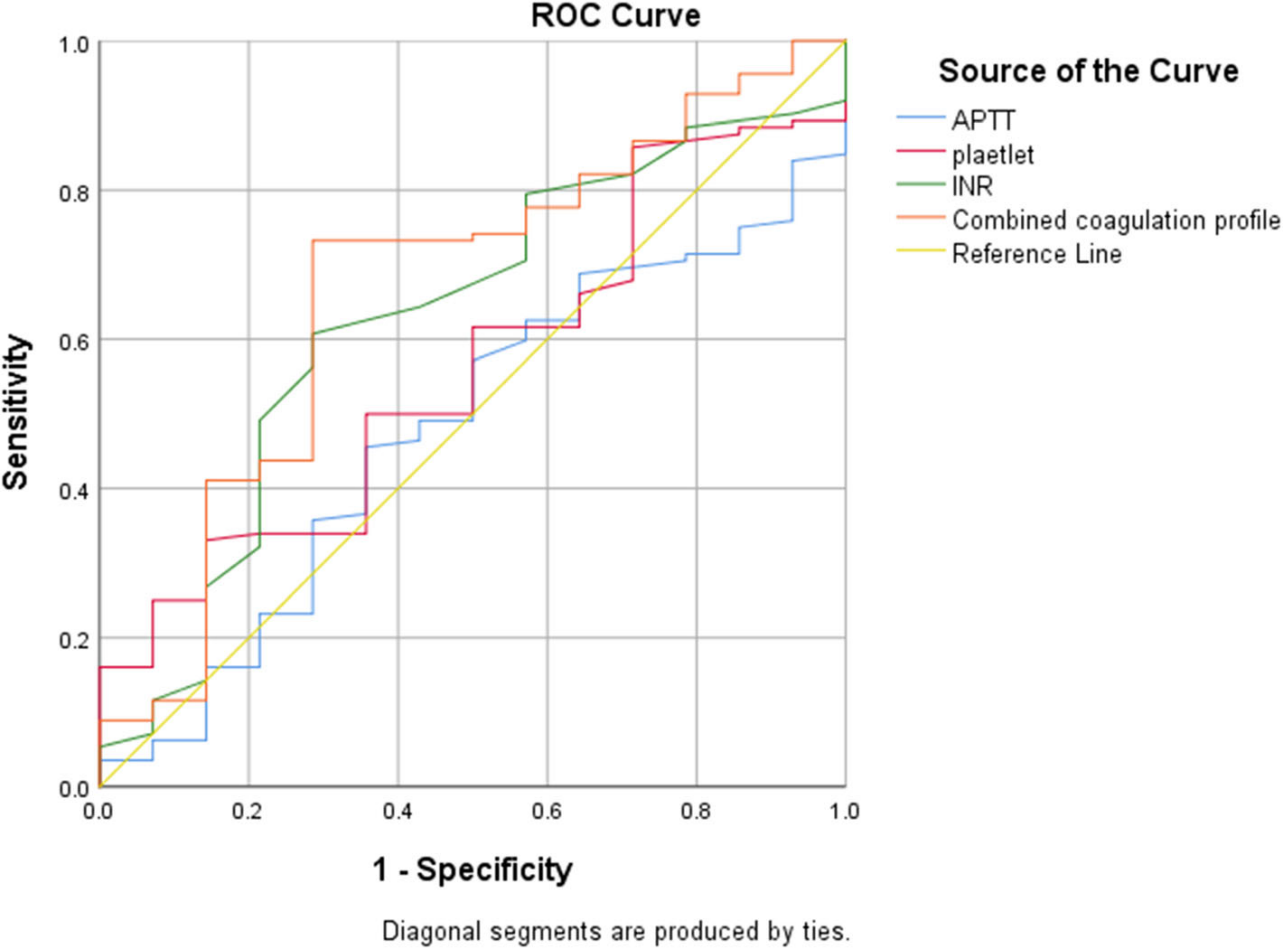
The diagnostic value of the coagulation profile evaluated in ROC curves

**Fig. 3:**
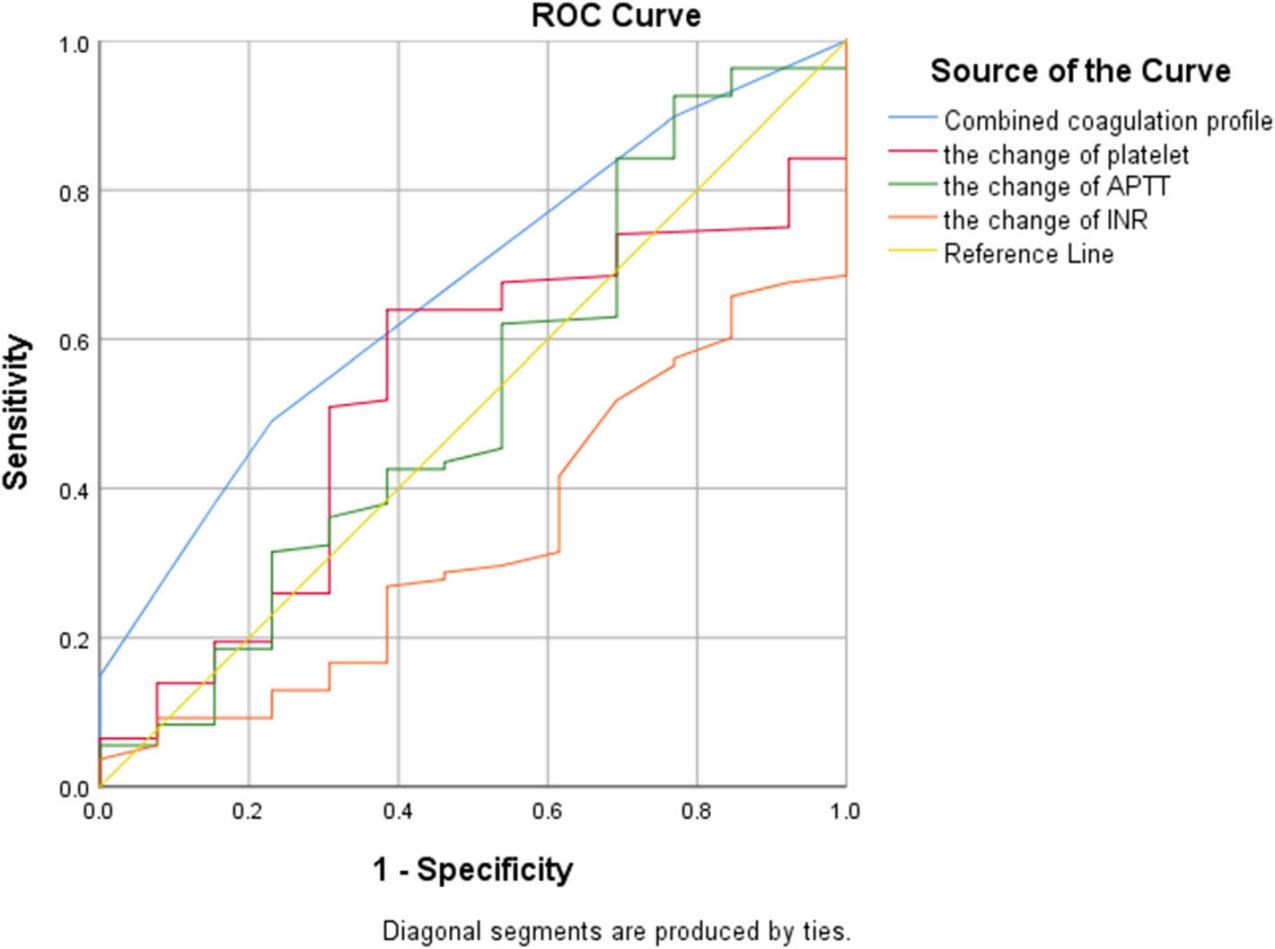
The diagnostic values of the change in coagulation profile from pre-resection to pre-implantation shown in ROC curves

The diagnostic value of the coagulation profile was evaluated in ROC curves (Fig. 2). The AUCs of plasma D-dimer, platelet count, APTT, TT, and INR before reimplantation for predicting persistent infection were 0.542 (0.359, 0.725), 0.560 (0.417, 0.702), 0.526 (0.377, 0.674), 0.482 (0.29, 0.674), and 0.632 (0.479, 0.786), respectively. Furthermore, the coagulation profile was combined by logistic regression (Additional file 1: Appendix 1.) based on significantly upregulated markers. The AUC of the combined coagulation profile was 0.667 (95% CI 0.511, 0.823). The diagnostic efficiency of the coagulation profile before re-implantation is depicted in Table 4. Besides, the diagnostic values of the change in coagulation profile from pre-resection to pre-implantation were shown in ROC curves (Fig. 3). The AUCs of ΔD-dimer, Δplasma fibrinogen, Δplatelet count, ΔAPTT, ΔPT, ΔINR, and ΔTT for infection eradication were 0.611 (0.403, 0.819), 0.549 (0.363, 0.736), 0.528 (0.374, 0.682), 0.546 (0.378, 0.714), 0.545 (0.392, 0.699), 0.626 (0.488, 0.765), and 0.493(0.327, 0.659), respectively. The AUC of the combined coagulation profile was 0.667 (0.526, 0.808). The diagnostic efficiency of the change in coagulation profile from pre-resection to pre-implantation is shown in Table 4. Besides, the change of coagulation profile was combined by logistic regression (Additional file 2: Appendix2).

**Table 4:** The association between the coagulation profile and treatment outcomes of two-staged arthroplasty. Corresponding cut-off and predictive values

	***AUC (95% CI)***	***Yonden index***	***Optimal cut-off***	***Sensitivity***	***specificity***
*The coagulation profile before reimplantation*					
***APTT***	0.526(0.377,0.674)	0.170	33.45	92.9	24.1
***PT***	0.608(0.454,0.762)	0.3	13.15	58.6	28.6
***TT***	0.482(0.29,0.674)	0.158	16.05	57.1	41.4
***D-dimer***	0.542(0.359,0.725)	0.262	1.19	66.2	60
***Platelet count***	0.560(0.417,0.702)	0.188	231.5	33	85.7
***INR***	0.632(0.479,0.786)	0.335	1.005	62.1	71.4
***ESR***	0.465(0.282,0.647)	0.083	31	8.3	100
***CRP***	0.615(0.427,0.803)	0.318	0.33	81.8	50
***Combined coagulation profile***	0.667(0.511,0.823)	NA	NA	NA	NA
*The change of coagulation profile from preresection to preimplantation*					
***APTT***	0.546 (0.378, 0.714)	0.145	-3.8	78.6	21.4
***PT***	0.545 (0.392, 0.699)	0.175	0.95	18.3	81.7
***TT***	0.493 (0.327, 0.659)	0.096	2.5	10	100
***D-dimer***	0.611 (0.403, 0.819)	0.278	-0.395	70.7	57.1
***Platelet count***	0.528 (0.374, 0.682)	0.248	56.5	63.3	61.5
***INR***	0.626 (0.488, 0.765)	0.235	0.05	57.1	66.4
***Plasma fibrinogen***	0.549 (0.363, 0.736)	0.227	0.365	79.8	57.1
***Combined coagulation profile***	0.667 (0.526, 0.808)	NA	NA	NA	NA

## Discussion

There is still a lack of comprehensive studies that evaluate the change of coagulation profile from reresection to re-implantation in two-staged arthroplasty up to now [3, 15, 16]. To address this problem, this study evaluated the change in coagulation profile during this period and the use of coagulation profile for predicting infection eradication in two-staged arthroplasty.

PJI pathogens can secret endotoxins and exotoxins by which stimulate immune cells to produce cytokines such IL-6, TNF- α, and IL-6 [5, 6]. These cytokines can impair the normal coagulation system subsequently. Parvizi et al. revealed that the INR in PJI patients is higher than that in aseptic loosening patients [6]. These studies indicated that PJI patients suffered from abnormal hypocoagulation. Our previous studies have also shown similar results. The changed coagulation cascade may be ascribed to PJI pathogens and return to relatively normal when the pathogens were eradicated. This research revealed that the levels of APTT, PT, INR, plasma fibrinogen, and platelet count before reimplantation were significantly lower than that of before re-resection. Unfortunately, no significant difference was detected when these variables were compared between the infection-controlled and persistent infection groups. This may be the result of a small number of persistent infection patients.

The goal of two-staged arthroplasty for PJI treatment is to eradicate infection. However, defining the time for re-implantation is still challenging and previous studies failed to identify serologic markers to guide the time for reimplantation [3, 16]. Therefore, we evaluate the efficiency of the coagulation profile for predicting persistent infection. We found that the levels of INR and APTT before re-implantation may predict infection eradication despite relatively poor efficiency. Moreover, we built a logistic model based on the change of INR, APTT, and platelet count. This model performed better than anyone alone and some commonly used serologic markers such as ESR and CRP in predicting persistent infection.

The levels of APTT, INR, and platelet count before re-implantation were significantly lower than those before spacer implantation. Therefore, we evaluated whether the change of these coagulation markers (ΔAPTT, ΔINR, and Δplatelet count) from pre-resection to pre-implantation could indicate infection eradication. The change of coagulation profile may help doctors guide the timing of re-implantation. In 14 recurrent PJI cases, 7 cases underwent elevated APTT, INR, and platelet count (ΔAPTT>0, ΔINR>0, and Δplatelet count>0). However, no statistical significance was detected between the recurrent rate in the elevated group (ΔAPTT>0, ΔINR>0 and Δplatelet count>0) and that of in the non-elevated group (ΔAPTT<0, ΔINR<0 and Δplatelet count<0) because the sample size of recurrent infection is relatively small.

Other studies used modified MSIS criteria or repeat cultures as the indicator of persistent infection [17, 18]. However, these methods were not used in this study because repeat cultures were fraught with high false negative rates, and modified MSIS criteria were of limited efficiency. Besides, no good criteria are accepted commonly as the standard of persistent infection before re-implantation. Therefore, we hold the opinion that the follow-up outcomes of PJI patients can be a relatively accurate indicator for infection eradication because many PJI criteria are built based on the follow-up results [18]. The relatively long follow-up period is one of our advantages over other studies.

A literature review suggested that many researchers failed to find accurate serological markers that indicated persistent infection such as ESR and CRP, and our results revealed similar results. Some synovial fluid indicators can predict persistent infection such as synovial WBC count and the percentage of PMN [3, 19]. These findings indicated that the local response to persistent infection was more accurate than the systemic response to the diagnosis of persistent infection. However, this study revealed that the combination of coagulation profiles was of comparable efficiency compared to that of reported synovial fluid indicators.

D-dimmer is a product of fibrin degradation and fibrinogen is the precursor of fibrin. But the fluctuation in plasma fibrinogen and D-dimer was not detected in the same direction. Then we reviewed the original medical data and found that many data about the levels of plasma D-dimer were not accessible because this test was not performed regularly in our center. Therefore, the statistical power is not strong enough to detect the difference in the levels of plasma D-dimer between pre-resection and pre-implantation because of the relatively small sample size compared to that of plasma fibrinogen.

**Fig. 4:**
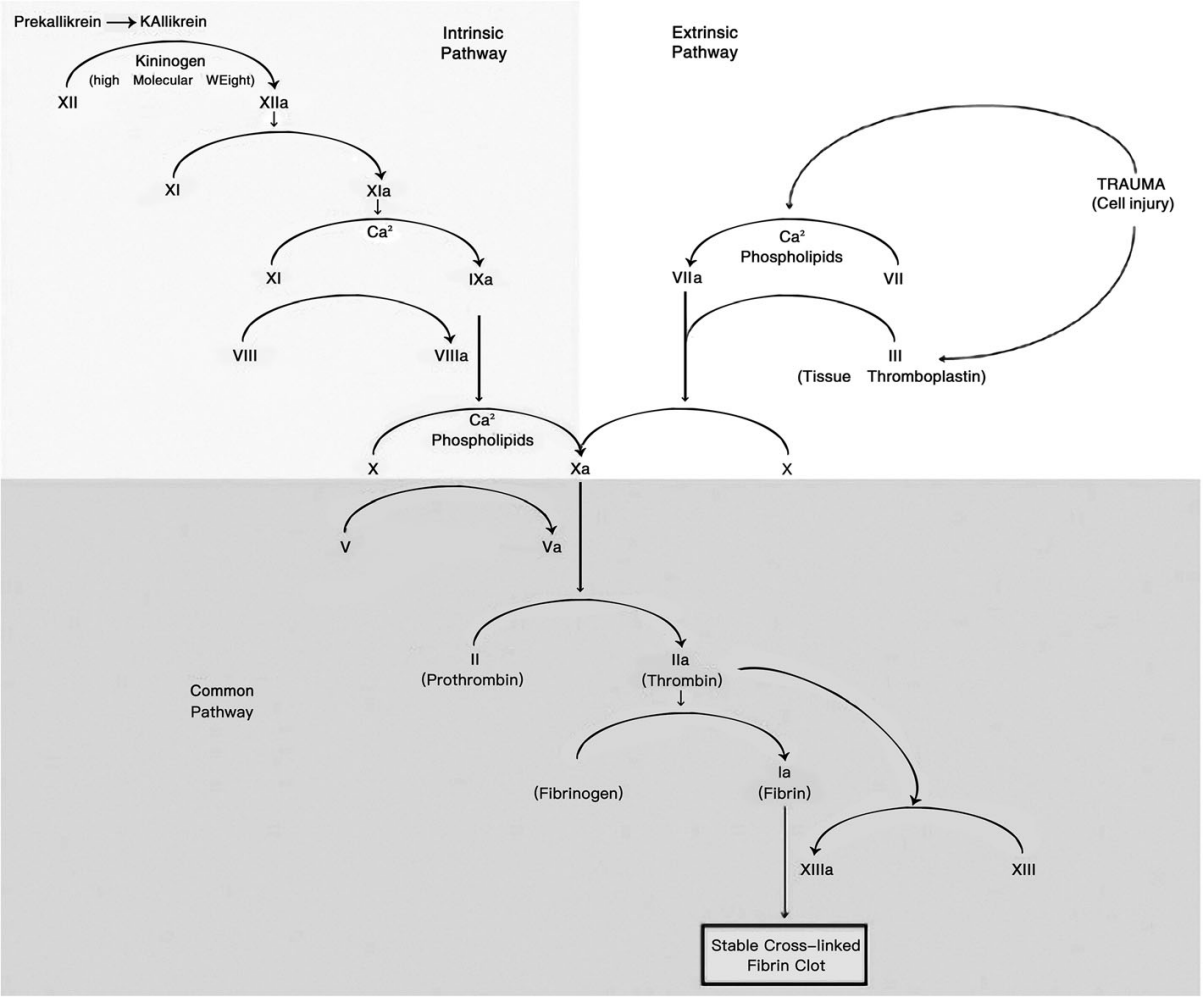
Part of intrinsic and extrinsic coagulation pathways’ changes

The levels of APTT and INR before pre-resection is higher than those before re-implantation. It suggests that intrinsic and extrinsic coagulation pathways changed before re-implantation compared to before pre-resection. Relatively robust TT revealed that the common pathway in the coagulation cascade did not change significantly between pre-resection and pre-implantation. However, as shown in Fig. 4 [6], part of intrinsic and extrinsic coagulation pathways’ changes significantly was not explored in this study. For example, which changed coagulation factor in intrinsic and extrinsic pathway cause the change of APTT and INR remains unknown. Moreover, this field needs further exploration.

We have shown that the coagulation profile may play a role in guiding the timing of implantation. The decrease in the levels of APTT and INR may indicate infection eradication and surgeons can take these markers into account before re-implantation. We believe that surgeons should continue to combine various diagnostic tools rather than a certain single marker to guide the timing of re-implantation, including the change of coagulation profile, serologic markers, clinical characteristics, and so on. For example, reduced APTT, INR, and platelet count before re-implantation compared to before pre-section may predict a higher success rate of re-implantation. How to identify the timing of re-implantation still needs to be further explored.

This study still has some limitations. First, two-staged arthroplasty is a relatively successful surgery and the rate of treatment failure is low in our center. Therefore, a large sample size is needed to reach statistical significance when comparing the difference of coagulation profile between the successful group and the persistent infection group. However, the sample size in this study is limited after strict inclusion and exclusion. Second, this study was performed in a tertiary center. The patients admitted to our joint center often suffered from severe infection and received previous treatments in other joint centers so that spacer exchange was not rare in this tertiary center. This fact can trigger a selection bias. Third, there is no gold standard for the diagnosis of infection eradication and we use follow-up results to evaluate infection eradication. However, some subclinical infections may be missed by patients or doctors during follow-up. This fact can trigger some biases. Finally, some patients underwent repeat debridement and spacer exchange before re-implantation. The decision of this was based on a combination of clinical appearance and laboratory tests. Besides, the patients who refused to receive re-implantation after spacer implantation and the patients who receive spacer implantation in other joint centers were excluded from this study. These conditions can also add bias to this study.

## Conclusion

The coagulation profile is different between pre-resection and pre-implantation in two-staged arthroplasty; the coagulation markers may play a role in predicting infection eradication before re-implantation when two-stage arthroplasty is performed.

## Supplementary Information

**Additional file 1: Appendix 1.**

**Additional file 2: Appendix 2.**

## Data Availability

All data and materials were in full compliance with the journal’s policy.

## Abbreviations

TJA: Total joint arthroplasty
PJI: Periprosthetic joint infection
MSIS: Musculoskeletal Infection Society criteria
PT: Prothrombin time
APTT: Activated partial thromboplastin time
INR: International normalized ratio
PTA: Prothrombin activity
TT: Thrombin time
