# Supplementary material for: The change of coagulation profile in two-staged arthroplasty for periprosthetic joint infection patients: a retrospective cohort study
Source: J Orthop Surg Res. 2021 May 18;16:319. doi: 10.1186/s13018-021-02477-4 (PMC8130413; doi:10.1186/s13018-021-02477-4)
Supplement: Supplementary file 1 — Additional file 1: Appendix 1. [file 13018_2021_2477_MOESM1_ESM.docx]

The logistic model combined by the coagulation profile before preimplantation.

Logit(P)=-3.05-0.005platelet+6.876INR-0.077APTT
