# Supplementary material for: The change of coagulation profile in two-staged arthroplasty for periprosthetic joint infection patients: a retrospective cohort study
Source: J Orthop Surg Res. 2021 May 18;16:319. doi: 10.1186/s13018-021-02477-4 (PMC8130413; doi:10.1186/s13018-021-02477-4)
Supplement: Supplementary file 2 — Additional file 2: Appendix 2. [file 13018_2021_2477_MOESM2_ESM.docx]

The logistic model combined by the change of coagulation profile between preresection and preimplantation.

Logit(P)=21.339-19.082Δplatelet-0.857ΔINR+0.4ΔAPTT
